# The Relationship between Cold Hypersensitivity in the Hands and Feet and Health-Related Quality of Life in Koreans: A Nationwide Population Survey

**DOI:** 10.1155/2019/6217036

**Published:** 2019-04-15

**Authors:** Kwang-Ho Bae, Youngseop Lee, Ho-Yeon Go, Su-Jung Kim, Si-woo Lee

**Affiliations:** ^1^Future Medicine Division, Korea Institute of Oriental Medicine, Daejeon, Republic of Korea; ^2^Department of Korean Internal Medicine, College of Korean Medicine, Se-Myung University, Chungju, Republic of Korea

## Abstract

**Aim:**

We investigated the distribution of cold hypersensitivity in the hands and feet (CHHF) and examined the association between CHHF and health-related quality of life (HRQOL) among Koreans.

**Methods:**

Stratified multistage sampling was used for random selection of 2,201 adults. HRQOL was assessed using the Short-Form 12-Item Health Survey (SF-12). Cold hypersensitivity was measured using a new self-report questionnaire to score the extent of cold sensation in their hands, feet, and abdomen using a 7-point scale. The correlation between CHHF and HRQOL was analysed using multiple regression analysis.

**Results:**

Cold hypersensitivity was present in the hands of 21.6%, the feet of 23.0%, and the abdomen in 22.5% of participants. Cold hypersensitivity in the hands and feet was observed in 17.9%, at least one body part (hands, feet, or abdomen) in 34.2%, and all three body regions in 12.3% of participants. The prevalence of cold hypersensitivity was significantly higher among women than among men, irrespective of the involved body part. Cold hypersensitivity scores in the hands and feet correlated negatively with body mass index, but not with age. The physical component summary (PCS) and mental component summary (MCS) of the SF-12 were both significantly lower in women with than in those without CHHF. Among men, only the PCS was significantly lower in the CHHF group. Multiple regression analysis, adjusted for sociodemographic variables, age, sex, and body mass index (BMI), confirmed that CHHF had negative effects on PCS and MCS.

**Conclusions:**

CHHF is more common in women and in individuals with a lower BMI. CHHF has an independent negative effect on HRQOL.

## 1. Introduction

Cold hypersensitivity in the hands and feet (CHHF) is the symptom of sensation of cold in the extremities under conditions that would not typically evoke such a sensation. CHHF is commonly observed in East Asians, especially women, and is a result of the abnormal contraction of blood vessels in the extremities [1, 2]. Although CHHF can be caused by neurological disorders, such as multiple sclerosis [3], metabolic disorders, such as hypothyroidism [4], cardiovascular disease [5], and adverse drug reactions [6], the majority of cases are idiopathic. Because the symptom is often accompanied by reduced peripheral circulation in the absence of arteriosclerosis, it is also sometimes referred to as primary vascular dysregulation [5, 7].

CHHF is considered more important in Korean medicine and other types of traditional East Asian medicine (TEAM) than in Western medicine. This is because CHHF is an important diagnostic factor in TEAM and is traditionally known to accompany several chronic symptoms and diseases, such as dyspepsia, menstrual pain, fatigue, and infertility [8, 9]. Therefore, in TEAM, CHHF is recognized as a symptom that needs to be treated proactively. Recently, several studies have demonstrated TEAM to be an effective treatment for CHHF. For example, one study in which Korean red ginseng was administered to individuals with CHHF found that the treatment significantly increased the skin temperature of the hands and feet in comparison to a placebo treatment [10]. Furthermore, a Japanese study reported that peripheral blood flow could be improved in a peripheral coldness group by administering traditional herbal medicines that are frequently used to treat CHHF [7].

Although the prevalence of CHHF and its effects on health-related quality of life (HRQOL) are important with regard to health care and policies, studies related to CHHF are insufficient. In Korea, one study investigated the location (hands, feet, abdomen, knees, etc.) and severity of cold hypersensitivity in 362 women visiting the gynaecology outpatient department of one university-affiliated hospital of Korean medicine [11]. Additionally, one Japanese study examined the relationship between cold hypersensitivity location, time of onset, family history, and the Cornell Medical Index (CMI) [12]. There have also been some related studies in Switzerland and the United States [13, 14]. There have been some studies on the characteristics and circumstances of CHHF patients, but these were either restricted to only patients, certain age groups, or women, or else were conducted in subjects from a country with different physiological and cultural characteristics than those of Koreans. Therefore, these studies have limitations for understanding the characteristics and distribution of CHHF in the Korean population.

Hence, this study aimed to investigate the prevalence of cold hypersensitivity by location (hands, feet, abdomen), and its effect on HRQOL, using a nationwide survey conducted over two rounds in 2013 and 2015.

## 2. Methods

### 2.1. Participants

The participants in this study were adult Korean men and women aged 19 years and over. The questionnaire survey was conducted by petition to a specialist organization (Gallup Korea Institute). Registered citizen population statistics were used for random extraction of participants using stratified multistage sampling based on region, sex, and age (95% confidence interval, sampling error ± 3.0%). A total of 1101 participants were surveyed between May 6 and June 14, 2013, and 1100 participants were surveyed between October 1 and October 30, 2015.

The survey was conducted through face-to-face interviews by a trained interviewer. The participants responded to structured questionnaires with the assistance of the interviewer. These questionnaires did not include the participants' personal information (address, citizen registration number, telephone number, name), and the participants provided verbal consent to participation after receiving an explanation of the objectives of the questionnaires and their use.

### 2.2. Ethics Approval and Consent to Participate

This survey did not collect personal information on participants. Participants verbally agreed that the questionnaire results could be used for research purposes. This study was approved by the Institutional Review Board of the Semyung University Korean Medicine Hospital (No. 1707-10).

### 2.3. Cold Hypersensitivity

The questionnaire about cold hypersensitivity required participants to respond to questions about their hands, feet, and abdomen in everyday life. Each question was scored on a 7-point scale, whereby a score closer to 7 indicated a colder condition, and a score closer to 1 indicated a warmer condition. A score of 5–7 points for one location was defined as cold hypersensitivity of that area, a score of 5–7 points in both the hands and the feet was defined as CHHF, while a score of 1–4 points in both the hands and the feet was defined as non-CHHF.

### 2.4. Health-Related Quality of Life

The participants' HRQOL was assessed using the Short-Form 12 Item Health Survey (SF-12 v2). The SF-12 is a health-related questionnaire with established validity and reliability. It is divided into the physical component summary (PCS) and the mental component summary (MCS) and consists of 8 domains. Scores range from 0 to 100 points, with a higher score indicating better health [15–17]. We also surveyed sociodemographic variables known to be associated with HRQOL, such as occupation, income, education, marital status, alcohol consumption, and smoking [18].

### 2.5. Statistical Analysis

Participants' general characteristics are expressed as the mean ± SD (standard deviation) and frequency. Chi-square tests and independent* t*-tests were used to compare data between different time points, cold hypersensitivity prevalence between males and females, and HRQOL scores and anthropometric variables according to CHHF status. The correlations of the cold hypersensitivity score with age and body mass index (BMI) were evaluated for each body part using Pearson's correlation coefficient. A multiple regression analysis was performed to verify whether CHHF acted as an independent factor affecting HRQOL after adjustment for sex, age, BMI, and sociodemographic variables, such as drinking, smoking, marital status, income, occupation, and education, as variables that could affect CHHF and HRQOL. A* P* value of less than 0.05 was considered statistically significant. SPSS 22.0 (IBM Corp., Armonk, NY, USA) was used for all statistical analyses.

## 3. Results

### 3.1. General Characteristics

There were 2201 participants in total, comprising 1090 men (49.5%) and 1111 women (50.5%). The mean age of participants was 46.5 ± 15.4 years and the mean BMI was 23.2 ± 2.9 kg/m^2^. Mean cold hypersensitivity scores were 3.4, 3.5, and 3.6 for the hands, feet, and abdomen, respectively. Other general characteristics are presented in Table 1.

### 3.2. Correlations of Cold Hypersensitivity with BMI and Age

Cold hypersensitivity scores in the hands and feet were strongly positively correlated, with correlation coefficients exceeding 0.8 in both men and women; there were also moderate positive correlations between cold sensitivity in the abdomen and the hands, and in the abdomen and the feet (correlation coefficients: 0.597, 0.638, respectively). Age did not correlate with cold hypersensitivity, but BMI was negatively correlated with cold hypersensitivity in the hands and feet. Abdominal cold hypersensitivity and BMI did not show a significant correlation in women or men, individually, but did show a weak negative correlation across all participants combined (Table 2).

### 3.3. Distribution of Cold Hypersensitivity

**Figure 1:**
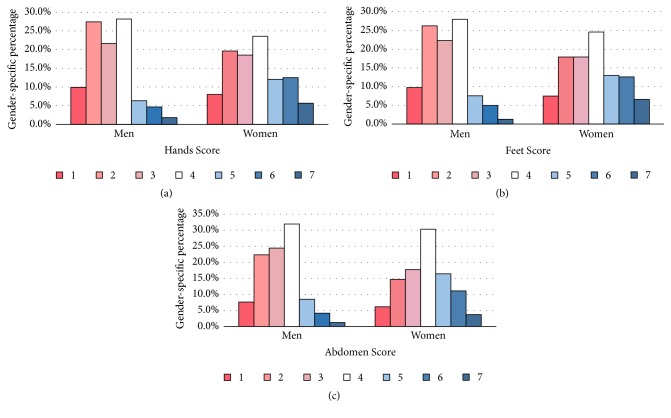
Distribution of response to cold hypersensitivity questionnaire by sex.* P* values were calculated using the chi-square test, all p < 0.001.

As shown in Figure 1, participants' cold hypersensitivity scores for the hands, feet, and abdomen all tended to be higher in women than in men, and these differences were significant (p < 0.001).

**Figure 2:**
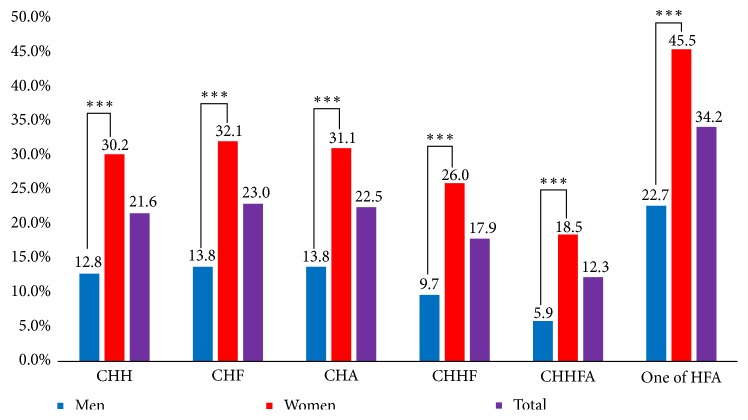
The prevalence of cold hypersensitivity by sex and body part. CHH: cold hypersensitivity in the hands; CHF: cold hypersensitivity in the feet; CHA: cold hypersensitivity in the abdomen; CHHF: cold hypersensitivity in the hands and feet; CHHFA: cold hypersensitivity in the hands, feet, and abdomen; one of HFA: cold hypersensitivity in just one of the three areas (hands, feet, or abdomen).* P* values were calculated using the chi-square test. ^*∗∗∗*^p < 0.001.

Cold hypersensitivity of the hands was observed in 21.6%, cold hypersensitivity of the feet was observed in 23.0%, and cold hypersensitivity of the abdomen was observed in 22.5% of participants. Cold hypersensitivity of all three body parts was observed in 17.9% of participants, and 34.2% of participants showed cold hypersensitivity in at least one of the three body parts. The prevalence of cold hypersensitivity was significantly higher in women than in men, irrespective of the body part (Figure 2).

### 3.4. Differences between the CHHF and Non-CHHF Groups

The two groups showed no difference in age, but weight and BMI were lower in the CHHF group for both men and women. PCS and MCS were significantly lower in the CHHF group than in the non-CHHF group for women. For men, PCS was significantly lower in the CHHF group, but there was no significant difference between these groups in terms of MCS (Table 3).

### 3.5. Effects of CHHF on HRQOL

In a multiple regression analysis investigating the effects of CHHF on PCS and MCS, CHHF was found to have a statistically significant negative effect on PCS and MCS in all models, including Model 1, which did not use adjustment variables, Model 2, which corrected for age and sex, Model 3, which corrected for age, sex, and BMI, and Model 4, which added sociodemographic variables such as drinking, smoking, marital status, income, occupation, and education (Table 4).

## 4. Discussion

Here, we investigated the prevalence and characteristics of CHHF among Koreans by administering a self-report questionnaire survey to typical Korean adults. Overall, our results have implications for both basic research and clinical applications and suggest that people with CHHF might have poor health.

CHHF is not only a major factor for diagnosing certain conditions, such as cold syndrome (*hanjeung*;* han zheng*; *寒證*) or yang deficiency (*yangheo*;* yang xu*; 陽虛) in traditional Korean medicine, but is also considered to be associated with various other symptoms and diseases. Recently, several studies have reported that individuals with CHHF have a higher prevalence of functional dyspepsia (vomiting, motion sickness, epigastric burning, postprandial fullness, nausea, epigastric pain, and bloating), while they have a lower prevalence of metabolic syndrome [19, 20].

In related work, studies in Japan have investigated subjects with a cold disorder called ‘hiesho', which is a similar concept to CHHF, and reported a higher frequency of symptoms such as shoulder stiffness, fatigue, neck stiffness, eyestrain, knee pain, and abdominal pain [21, 22]. Moreover, in Flammer syndrome, which is characterized by primary vascular dysregulation and cold extremities, a higher frequency of normal tension glaucoma, low blood pressure, reduced thirst, tinnitus, and muscle cramps has been reported [23, 24]. One study on sleep and peripheral temperature claimed that the distal-proximal skin temperature gradient is closely related to the onset of sleep, and that problems with the peripheral circulation are associated with somatic illness [25].

Therefore, the public health sector is increasingly recognizing the importance of ascertaining the prevalence and distribution of CHHF. In one study of 3067 elderly individuals aged 65 years or older, 28.6% of women and 23.6% of men were reported to have cold feet and/or legs [26]. Another study examining 6998 black and white individuals aged 18 years or older reported that approximately 12% of subjects experienced the symptom of unusual sensitivity to cold in the fingers [13]. In Japan, which has similar cultural and ethnic characteristics to Korea, a study of 334 healthy individuals aged 50 years or older found that the proportion of subjects with sensitivity to cold varied with age and ranged from 6.1% to 30.8% for men and 14.3% to 28.3% for women [27]. A study of 318 women aged 20–51 years in Japan reported that 38.1% of their participants had experienced cold disorder [12].

In Korea, even though a large number of individuals visit medical institutions for CHHF, few studies have investigated the proportion of Koreans with CHHF or other cold disorders, and on the impact of CHHF on HRQOL. Indeed, the majority of studies of cold disorder or CHHF in Korea have only examined particular subject groups [11, 28], making it difficult to assess the distribution of CHHF throughout the general population. Thus, the prevalence and characteristics of CHHF and its effects on HRQOL in this population has not been reported to date. Although this study was able to show the distribution of cold hypersensitivity of the hands, feet, and abdomen among Koreans, the findings cannot be accurately compared with cold hypersensitivity in other ethnicities, since the instruments and questionnaire content differed from those used in previous studies. Nevertheless, compared to the prevalence of 12% reported for cold hypersensitivity of the hands in Caucasian and African subjects, the present study found a prevalence of 21.6%. Taken together with previous studies, we believe that the prevalence of CHHF is higher in Koreans and other East Asians than in other ethnicities [13, 29]. Perhaps because of the high frequency of CHHF in East Asia, it seems that CHHF is receiving more attention in East Asia than in other regions.

As previously mentioned, at an institution of traditional Korean medicine, subjects most frequently complained of cold sensation in their hands, feet, and abdomen [11]. Hence, we included questions on temperature sensation in these three body parts in our questionnaire. In order to evaluate HRQOL, we used the SF-12, which has recognized reliability and validity worldwide, and is able to assess physical and mental components [11, 15–17].

As shown in Figure 1 and Table 2, this study elucidated the correlations between cold hypersensitivity scores, BMI, and age, as well as the distribution of cold hypersensitivity scores by sex. Consistent with previous studies, we found that a lower BMI was associated with more severe cold hypersensitivity in the hands and feet, and that cold hypersensitivity scores were higher in women than in men [14, 27, 30, 31]. However, abdominal cold hypersensitivity showed no correlation with BMI when analysed by sex but showed a very weak negative correlation when all participants were included in the analysis. Another study observed that an obese group with high BMI showed higher temperatures in their fingers, but a significantly lower abdominal temperature [32]. Based on this result, we believe that the actual temperature in the abdomen needs to be approached differently than assessing a cold sensation or related discomfort and that further studies on this subject are required.

Age showed no correlation with cold hypersensitivity scores in either men or women; this differs from the results of Mozaffarieh and colleagues, who reported a negative correlation between CHHF and age in women [15]. However, the methodology used by Mozaffarieh et al. [15] differs from our own; they used regression analysis, whereas we used correlation analysis. Since a study in Japan also found that cold disorder was frequently observed not only during climacterium, but also in young women of about 20 years old, additional studies on the relationship between CHHF and age are required.

Using the SF-12, the relationship between CHHF and HRQOL was evaluated in terms of the MCS and PCS. Since previous studies had confirmed a high prevalence of various symptoms and diseases in groups with CHHF, we predicted that similar trends would be observed in the general Korean population [12, 19, 21, 23]. As shown in Table 3, the CHHF group showed significantly lower scores for PCS among men and for MCS and PCS among women. Moreover, Table 4 shows that even after adjusting for age, sex, BMI, and sociodemographic variables, CHHF had an independent negative effect on MCS and PCS. In particular, as shown in Table 3, the difference in mean scores between the CHHF group and the non-CHHF group was larger in women, and MCS was only significantly different between the two groups for women. This suggests that CHHF has a greater impact on HRQOL in women than in men, and therefore women need active management of CHHF.

This study has the following limitations. First, because the questionnaire used in this study only assesses the severity of cold sensation in each body part, participants may have differed in their standards for discomfort related to cold hypersensitivity. A more objective instrument will need to be developed in the future to overcome this problem. In addition, because we were unable to survey the duration of symptoms and the time of onset, it was not possible to evaluate the impact of CHHF duration on HRQOL. Second, it is difficult to directly compare the results of this study with those of previous studies, since previous CHHF studies used different evaluation and diagnostic methods. Third, because CHHF is influenced by race, culture, and the environment [1, 30], it is difficult to apply the result of this study to population groups in other countries. Another potential limitation is that the surveys for this study were conducted in two rounds, in May 2013 and October 2015; the mean temperature difference between these two time points was approximately 3°C (May 2013, nationwide average temperature 17.8°C, average maximum temperature 24.1°C, average minimum temperature 12.1°C; October 2015, nationwide average temperature 15.0°C, average maximum temperature 21.5°C, average minimum temperature 9.6°C) [33, 34]. However, as can be seen in Table 1, this temperature difference did not produce a difference in cold hypersensitivity scores for the hands, feet, or abdomen, and therefore cannot be considered as a confounding variable.

Despite these limitations, this study differs from previous studies that have largely investigated patients at medical institutions. Specifically, we recruited a sample analogous to the general Korean population by stratifying participants by age, sex, and region. Therefore, our study was able to examine the distribution of cold hypersensitivity in Korea more accurately compared with any previous study, so the data on tendency and distribution of CHHF in Koreans collected through this study can be used as a reliable basis for preparing health policies and medical care guidelines for this symptom in the future. In addition, the results of this study on the relationship between CHHF, BMI, and HRQOL suggest that people with CHHF need management of CHHF in order to improve HRQOL, and one of the ways to improve the HRQOL is to avoid low BMI.

## 5. Conclusion

In conclusion, this study elucidated the distribution of cold hypersensitivity among Korean adults by body part and sex and also demonstrated a clear negative correlation between BMI and CHHF. Finally, we confirmed that CHHF had an independent negative effect on PCS and MCS, even after adjusting for age, sex, BMI, and sociodemographic variables.

## Figures and Tables

**Table 1:** Participants' general characteristics.

Category	2013	2015	P-value	Total
Sex				
Male	545	545	0.983	1090 (49.5)
Female	556	556		1111 (50.5)
Age (years)				
20–29	183 (16.6)	194 (17.6)	0.852	377 (17.1)
30–39	224 (20.3)	204 (18.5)		428 (19.4)
40–49	243 (22.1)	234 (21.3)		477 (21.7)
50–59	217 (19.7)	218 (19.8)		435 (19.8)
60–69	120 (10.9)	130 (11.8)		250 (11.4)
Over 70	114 (10.4)	120 (10.9)		234 (10.6)
Mean	46.4 ± 15.3	46.6 ± 15.6	0.731	46.5 ± 15.4
Body mass index (kg/m^2^)	23.2 ± 3.0	23.2 ± 2.8	0.937	23.2 ± 2.9
Drinking^*∗*^				
Current drinker	961 (87.3)	720 (65.5)	< 0.001	1681 (76.4)
Not drinking	140 (12.7)	380 (34.5)		520 (23.6)
Smoking				
Current smoker	293 (26.6)	275 (25.0)	0.387	568 (25.8)
Not smoking	808 (73.4)	825 (75.0)		1633 (74.2)
Marital status				
With spouse	814 (74.0)	777 (70.6)	0.078	1591 (72.3)
Without spouse	286 (26.0)	323 (29.4)		609 (27.7)
Income^†^				
Under 200	199 (18.2)	142 (12.9)	0.001	341 (15.5)
200–300	219 (20.0)	187 (17.0)		406 (18.5)
300–400	299 (27.3)	325 (29.5)		624 (28.4)
400–500	167 (15.3)	184 (16.7)		351 (16.0)
Over 500	211 (19.3)	262 (23.8)		473 (21.5)
Occupation^$^				
Office	219 (19.9)	253 (23.0)	0.130	472 (21.4)
Service	390 (35.4)	394 (35.8)		784 (35.6)
Others	492 (44.7)	453 (41.2)		945 (42.9)
Education				
≤ Middle school	208 (19.0)	162 (14.7)	0.017	370 (16.9)
High school	420 (38.4)	466 (42.4)		886 (40.4)
≥ University	466 (42.6)	472 (42.9)		938 (42.8)
Hands score	3.5 ± 1.6	3.4 ± 1.5	0.413	3.4±1.6
Feet score	3.5 ± 1.6	3.5 ± 1.5	0.973	3.5±1.6
Abdomen score	3.6 ± 1.5	3.6 ± 1.4	0.627	3.6±1.4

Total	1101 (50.0)	1100 (50.0)		2201 (100)

Data are shown as n (%) or the mean ± standard deviation. P values were calculated by chi-square test or Student's *t*-test

^*∗*^  “Current drinker” was defined as alcohol consumption at least once a month; “Not drinking” was defined as alcohol consumption less than once a month.

^†^  Average monthly household income (unit: 10,000 KRW)

^$^  “Office” includes administrative, professional, and clerical work; “Service” includes service and sales industries; “Others” includes skilled or machine work, labour, agriculture and forestry, fishing, and unemployment.

**Table 2:** Correlations between cold hypersensitivity scores, body mass index, and age, by sex and affected body part.

	Hands	Feet	Abdomen	BMI	Age
Men					
Hands	1				
Feet	0.805^*∗∗*^	1			
Abdomen	0.580^*∗∗*^	0.637^*∗∗*^	1		
BMI	-0.118^*∗∗*^	-0.110^*∗∗*^	-0.057	1	
Age	0.037	0.028	0.002	0.059	1

Women					
Hands	1				
Feet	0.848^*∗∗*^	1			
Abdomen	0.583^*∗∗*^	0.614^*∗∗*^	1		
BMI	-0.182^*∗∗*^	-0.162^*∗∗*^	-0.052	1	
Age	-0.014	-0.006	0.031	0.393^*∗∗*^	1

Total					
Hands	1				
Feet	0.837^*∗∗*^	1			
Abdomen	0.597^*∗∗*^	0.638^*∗∗*^	1		
BMI	-0.189^*∗∗*^	-0.179^*∗∗*^	-0.095^*∗∗*^	1	
Age	0.021	0.023	0.031	0.220^*∗∗*^	1

BMI, body mass index; ^*∗∗*^p < 0.01.

**Table 3:** Difference in general characteristics between CHHF and non-CHHF groups.

	CHHF	Non-CHHF	P-value
Men			
N	106	906	
Age	48.0 ±15.7	45.2 ±14.9	0.071
Height	172.0 ±5.7	172.5 ±5.4	0.390
Weight	68.5 ±8.2	71.2 ±9.2	0.003
BMI	23.1 ±2.4	23.9 ±2.6	0.004
PCS	51.3 ±6.7	52.7±6.1	0.024
MCS	50.4 ±7.5	51.2 ±7.9	0.282
Drinking^*∗*^			
Current drinker	93 (87.7)	789 (87.1)	0.850
Not drinking	13 (12.3)	117 (12.9)	
Smoking			
Current smoker	43 (43.4)	439 (48.5)	0.324
Not smoking	60 (56.6)	467 (51.5)	
Marital status			
With spouse	77 (72.6)	636 (70.2)	0.602
Without spouse	29 (27.4)	270 (29.8)	
Income^†^			
Under 200	23 (21.7)	121 (13.4)	0.174
200–300	19 (17.9)	180 (19.9)	
300–400	28 (26.4)	270 (29.8)	
400–500	13 (12.3)	150 (16.6)	
Over 500	23 (21.7)	184 (20.3)	
Occupation^$^			
Office	22 (20.8)	251 (27.7)	0.088
Service	36 (34.0)	339 (37.4)	
Others	48 (45.3)	316 (34.9)	
Education			
≤ Middle school	17 (16.0)	96 (10.6)	0.211
High school	35 (33.0)	343 (37.9)	
≥ University	54 (50.9)	466 (51.5)	
Women			
N	289	707	
Age	47.1 ±16.2	47.6 ±15.3	0.688
Height	158.8 ±5.3	159.2 ±5.0	0.247
Weight	55.3 ±6.9	57.5 ±7.4	< 0.001
BMI	22.0 ±2.9	22.7 ±3.0	< 0.001
PCS	48.1 ±9.4	50.6 ±7.7	< 0.001
MCS	48.2 ±8.7	50.8 ±8.3	< 0.001
Drinking^*∗*^			
Current drinker	201 (69.6)	457 (64.6)	0.078
Not drinking	88 (30.4)	250 (35.4)	
Smoking			
Current smoker	11 (3.8)	16 (2.3)	0.127
Not smoking	278 (96.2)	691 (97.7)	
Marital status			
With spouse	211 (73.3)	537 (76.0)	0.373
Without spouse	77 (26.7)	170 (24.0)	
Income^†^			
Under 200	56 (19.5)	109 (15.5)	0.458
200–300	52 (18.1)	121 (17.2)	
300–400	74 (25.8)	200 (28.4)	
400–500	40 (13.9)	118 (16.7)	
Over 500	65 (22.6)	157 (22.3)	
Occupation^$^			
Office	61 (21.1)	100 (14.1)	0.025
Service	89 (30.8)	243 (34.4)	
Others	139 (48.1)	364 (51.5)	
Education			
≤ Middle school	58 (20.1)	162 (23.1)	0.074
High school	112 (38.9)	306 (43.6)	
≥ University	118 (41.0)	234 (33.3)	
Total			
N	395	1613	
Age	47.3 ± 16.1	46.2 ± 15.1	0.191
Height	162.3 ± 8.0	166.6 ± 8.4	< 0.001
Weight	58.8 ± 9.3	65.2 ± 10.8	< 0.001
BMI	22.3 ± 2.8	23.4 ± 2.9	< 0.001
PCS	48.9 ± 8.9	51.8 ± 6.9	< 0.001
MCS	48.8 ± 8.4	51.1 ± 8.1	< 0.001
Drinking^*∗*^			
Current drinker	294 (74.4)	1246 (77.2)	0.235
Not drinking	101 (25.6)	367 (22.8)	
Smoking			
Current smoker	57 (14.4)	455 (28.2)	< 0.001
Not smoking	338 (85.6)	1158 (71.8)	
Marital status			
With spouse	288 (73.1)	1173 (72.7)	0.881
Without spouse	106 (26.9)	440 (27.3)	
Income^†^			
Under 200	79 (20.1)	230 (14.3)	0.035
200–300	71 (18.1)	301 (18.7)	
300–400	102 (26.0)	470 (29.2)	
400–500	53 (13.5)	268 (16.6)	
Over 500	88 (22.4)	341 (21.2)	
Occupation^$^			
Office	83 (21.0)	351 (21.8)	0.147
Service	125 (31.6)	582 (36.1)	
Others	187 (47.3)	680 (42.2)	
Education			
≤ Middle school	75 (19.0)	258 (16.1)	0.295
High school	147 (37.3)	649 (40.4)	
≥ University	172 (43.7)	700 (43.6)	

CHHF, cold hypersensitivity in the hands and feet; BMI, body mass index; PCS, SF-12 Physical component summary; MCS, SF-12 mental component summary.

^*∗*^  “Current drinker” was defined as alcohol consumption at least once a month; “Not drinking” was defined as alcohol consumption less than once a month.

^†^  Average monthly household income (unit: 10,000 KRW)

^$^  “Office” includes administrative, professional, and clerical work; “Service” includes service and sales industries; “Others” includes skilled or machine work, labour, agriculture and forestry, fishing, and unemployment.

**Table 4:** Multiple regression analysis for the association of CHHF and HRQOL.

Models	Dependent variable (SF-12)							
	PCS				MCS			
	B	SE	*β*	*P* value	B	SE	Β	*P* value
Model 1	-2.889	0.42	-0.15	< 0.001	-2.328	0.46	-0.11	< 0.001
Model 2	-2.070	0.38	-0.11	< 0.001	-2.094	0.47	-0.10	< 0.001
Model 3	-2.163	0.39	-0.12	< 0.001	-2.098	0.47	-0.10	< 0.001
Model 4	-2.144	0.38	-0.11	< 0.001	-2.096	0.48	-0.10	< 0.001

CHHF, cold hypersensitivity in the hands and feet; HRQOL, health-related quality of life; Model 1: unadjusted; Model 2: adjusted for gender and age; Model 3: adjusted for gender, age, and body mass index; Model 4: adjusted for gender, age, body mass index, and all sociodemographic variables, such as drinking, smoking, marital status, income, occupation, and education; B, unstandardized regression coefficients; SE, standard error of unstandardized coefficients; *β*, standardized regression coefficients; PCS, SF-12 physical component summary; MCS, SF-12 mental component summary.

## Data Availability

The data that support the findings of this study are available from the Korean medicine Data Center (KDC, http://kdc.kiom.re.kr), but restrictions apply to the availability of these data, which were used under license for the current study and therefore are not publicly available. However, the data are available from the KDC for researchers who meet the criteria for access to confidential data.
